# Supplementary material for: New endophytic strains of Trichoderma promote growth and reduce clubroot severity of rapeseed (Brassica napus)
Source: PLoS One. 2023 Oct 31;18(10):e0287899. doi: 10.1371/journal.pone.0287899 (PMC10617699; doi:10.1371/journal.pone.0287899)
Supplement: S1 File — (DOCX) [file pone.0287899.s001.docx]

**S1 Table. Primer pairs used for examining the expression of defense genes using qPCR**

| **Gene name** | **Primer pairs** | **Reference** |
| --- | --- | --- |
| *BnPR*-1 | BnPR-1 F: 5′-ATGAAAGTCACTAACTGTTCTCGAC-3′  BnPR-1 R: 5′-GCCAGTAAACTAGGTAACGGATAA-3′ | [35] |
| *BnPR*-2 | BnPR-2 F: 5′-GTGTTAGCATCATCACCAATGTTGCTG-3′  BnPR-2 R:5′-GGAGATTAGTTAAACTTAACACCATATTTAAGCTG-3′ | [35] |
| *BnPR*-5 | BnPR-5 F: 5′-CAATGGCTTCACGAAACCTCTTCAACTTCG-3′  BnPR-5R: 5′-GTGATTTTAACGGCGATGGTGAGGGCAAAA-3′ | [35] |
| *BnOPCL* | BnOPCL F: 5′-GTACAAGAGGGTACGAAAAGTGG-3′  BnOPCL R: 5′-TGGGTAAACCATCCTCAGG-3′ | [36] |
| *BnCCR* | BnCCR F: 5′-AGCCGAAAAGCTGCTTGGAG-3′  BnCCR R: 5′-TAGCCAACCGATTCACATCC-3′ | [36] |
| *BnACO* | BnACO F: 5′-CATTCTACAACCCTGGAAGCGAC-3′  BnACO R : 5′-ATGGTCCAACATTGTTGGCCAC-3′ | [36] |
| *BnSAM3* | BnSAM3 F: 5′-AGCTACGGAATGGGAAGATAC-3′  BnSAM3 R: 5′-AAGACCTGAGAGAACTAAAC-3′ | [36] |
| *BnOPRA2* | BnOPRA2 F: 5′-TGTCCTCACTTGCTACCGATGAGG-3′  BnOPRA2 R: 5′-GGATCAGAAGTGTAAAACGTTGGC-3′ | [36] |
| *BnAA01* | BnAA01 F: 5′-GAATGGAACATCCACCAGAAGAAG-3′  BnAA01 R : 5′-TGTGCTCATGTCATGTCTG-3′ | [36] |
| Actin | BnActin F: 5′-AAGAGCAGTTCTTGGGTGGA-3′  BnActin R: 5′-TACTTCAGGGCAACGGAATC-3′ | [36] |


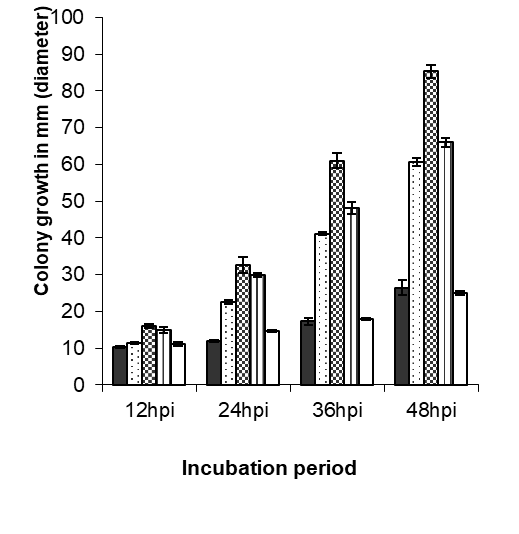

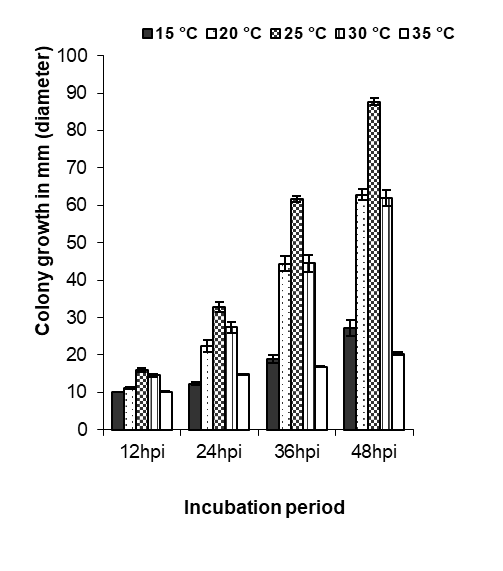


**A**

**B**

**S1 Fig**. **Growth of *Trichoderma* spp. (ReTk1 and ReTv2) at different temperatures range (A), ReTk1 and (B), ReTv2.**


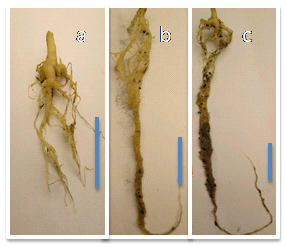


**S2 Fig. Development of new lateral roots in *Plasodiophora brassicae*-infected rapeseed plants after treatment with *Trichoderma* spp. (a) Without treatment, (b) and (c) treatment with ReTk1 and ReTv2.**
